# Dolphin Morbillivirus Epizootic Resurgence, Mediterranean Sea

**DOI:** 10.3201/eid1403.071230

**Published:** 2008-03

**Authors:** Juan-Antonio Raga, Ashley Banyard, Mariano Domingo, Mandy Corteyn, Marie-Françoise Van Bressem, Mercedes Fernández, Francisco-Javier Aznar, Thomas Barrett

**Affiliations:** * University of Valencia, Valencia, Spain; † Institute for Animal Health, Pirbright, UK; ‡ Autonomous University of Barcelona, Bellaterra, Barcelona, Spain; § Museo de Delfines, Pucusana, Peru

**Keywords:** morbillivirus, dolphin, re-emerging disease, dispatch

## Abstract

In July 2007, >100 striped dolphins, *Stenella coeruleoalba*, were found dead along the coast of the Spanish Mediterranean. Of 10 dolphins tested, 7 were positive for a virus strain closely related to the dolphin morbillivirus that was isolated during a previous epizootic in 1990.

An epizootic caused by a newly recognized member of the genus *Morbillivirus,* the dolphin morbillivirus (DMV), killed thousands of striped dolphins (*Stenella coeruleoalba*) in the Mediterranean Sea during 1990–1992 (*1*–*4*). The first affected dolphins were detected in the Gulf of Valencia (Spanish Mediterranean) in July 1990, but the die-offs soon extended to other regions (*2*). Results of a serologic survey of *S. coeruleoalba* from the Gulf of Valencia and adjacent waters indicated that only adult dolphins harbored antibodies against DMV and that seroprevalence in this age class had decreased from 100% in 1990–1992 to 50% in the study (*5*). This finding suggests that the virus was not endemic, that the dolphins were losing their humoral immunity, and that the population was susceptible to new epizootics. The possibility of new epizootics was also supported by population data (*6*). The density of striped dolphins estimated in the Gulf of Valencia (0.49 dolphin/km^2^) in 2001–2003 was again close to the maximum reported for this species in the western Mediterranean (*6*). This high population density was likely to favor the propagation of morbillivirus infections (*7*).

## The Study

A new outbreak of die-offs among striped dolphins was detected in the Gulf of Valencia in early July 2007. At the time of writing, unusual die-offs had also been recorded in dolphins from the southern coasts of the Spanish Mediterranean, Balearic Islands, Catalonia, and the Ligurian Sea. Between July and October 2007, >100 dolphins had been found stranded along the Spanish Mediterranean coast. Carcasses were in different states of decomposition. Some dolphins were stranded alive; all had neurologic symptoms and died after being rescued.

**Figure 1:**
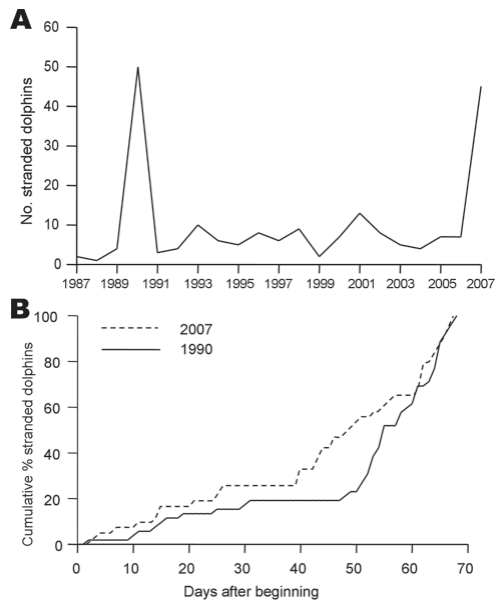
Stranding patterns of Mediterranean striped dolphins, *Stenella coeruleoalba*, in the Gulf of Valencia and adjacent waters (Valencian Community) (514 km of coastline). A) Records of *S. coeruleoalba* stranded in July–August each year from 1987 to 2007. Note peaks of stranding in 1990 and 2007. B) Cumulative percentages of dolphins found dead during July–August, 1990 and 2007 epizootics. Day 0 corresponds to June 23.

In the Gulf of Valencia, the number of stranded animals during July through August 2007 was similar to that recorded in 1990 during the same months (Figure 1, **panel A**). Stranding rate was also similar during each episode, with an initial low rate at the beginning of July, ≈2 weeks with no stranded dolphins reported, and then a sharp increase of stranding in mid-August (Figure 1, **panel B**). The most apparent difference between each episode was the size of animals collected: the mean size ± SD of dolphins collected in 1990 and 2007 was 180.9 ± 28.6 cm (n = 34) and 159.9 ± 40.9 cm (n = 17), respectively. This difference is statistically significant (Student *t* test, *t* = 2.14, 49 df, p = 0.037).

We examined 10 dolphins (5 adults and 5 juveniles) immediately after their death and collected samples of brain, lung, spleen, liver, and lymph nodes for histologic and molecular studies. Immunohistochemical examination for DMV antigen was performed. A monoclonal antibody to canine distemper virus (MoAb CDV-NP, VMRD, Inc., Pullman, WA, USA), known to react with DMV, was used as primary antiserum at a dilution of 1:200. The secondary antibody, a biotinylated goat anti-mouse immunoglobulin serum, was used at the same dilution. Finally, the avidin-biotin peroxidase complex was incubated at a dilution of 1:100. Sections were counterstained with hematoxylin.

**Figure 2:**
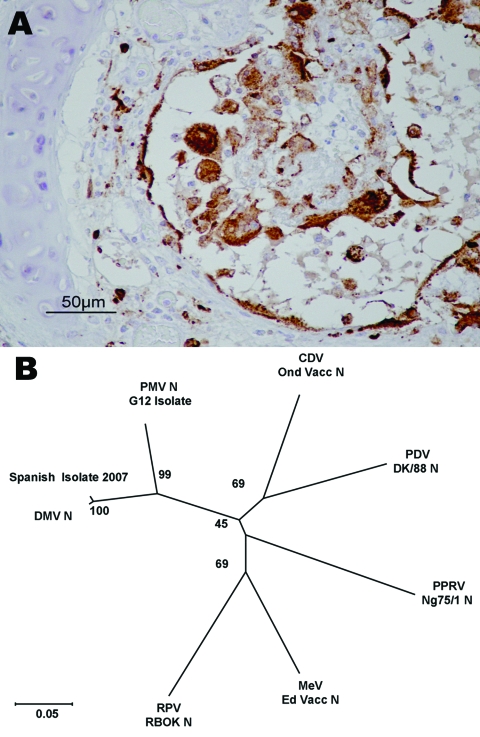
Evidence concerning the identity of the morbillivirus infecting Mediterranean striped dolphins in 2007. A) Immunohistologic staining of lung tissue with a MoAb anti CDV-NP, counterstained with hematoxylin. Epithelial bronchiolar cells and cells in the bronchiolar lumen are positively stained. B) Phylogenetic analysis of the N1/N2 region of the morbillivirus N genes. The number indicated at each node represents the bootstrap value after 10,000 replicates. The evolutionary distances were computed with the Kimura 2-parameter method and are in the units of the number of base substitutions per site. The tree is drawn to scale, with branch lengths in the same units as those of the evolutionary distances used to infer the phylogenetic tree. Details of each isolate used for this study and accession numbers for each of the sequences are as follows: DMV 2007 isolate (EU124652), PMV G12 isolate (AY949833), PPRV Nigeria/75/1 Vaccine (X74443), PDV DK/88 strain (X75717), RPV RBOK vaccine strain (Z30697), DMV isolate (AJ608288), CDV Onderstepoort vaccine strain (AF305419), and MV Edmonston vaccine strain (AF266288). DMV, dolphin morbillivirus; PMV, porpoise morbillivirus; PPRV, peste des petits ruminants virus; PDV, phocine distemper virus; RPV RBOK, rinderpest virus RBOK strain; CDV, canine distemper virus; MV, measles virus.

Lesions comprised multifocal bronchiolo-interstitial pneumonia with syncytial cells and nuclear inclusions in the alveolar epithelium and syncytia, as well as lymphoid depletion of the cortical area of the lymph nodes. In 1 dolphin, diffuse pyogranulomatous pneumonia of probable bacterial origin, with disseminated foci to many organs (e.g., brain, heart, lymph nodes, spleen), was found concomitantly with DMV infection. For 5 dolphins, immunostaining was observed in epithelial cells and syncytia in the lungs (Figure 2, **panel A**), lymphoid cells, neurons, and/or bile duct epithelium. The staining pattern was similar to that described for animals affected by the 1990 epizootic (*8*).

Tissues from the 3 dolphins analyzed by immunohistochemistry were also examined for morbillivirus nucleic acid by reverse transcription–PCR (RT-PCR). We used nested sets of universal morbillivirus primers (UPN1: 5′-ACAAACCNAGRATTGCTGAAATGAT-3′ [genome position 844–869]; UPN2: 5′-CTGAAYTTGTTCTGAAYTGAGTTCT-3′ [position 1057–1081]) based on the conserved N terminus of the morbillivirus N gene. Tissue samples from 1 dolphin tested positive by N1/N2, and the product was sequenced in its entirety. PCR with nested primers (N1a: 5′-ACTATYAARTTYGGNATNGAACNATGT-3′ [position 906–932]; N2a: 5′-ctgcactraayttgttytgrayngagt-3′ [position 1044–1071] confirmed the other samples as positive.

The N1/N2 sequence (European Molecular Biology Laboratory accession no. EU124652) was aligned to the same region for each of the morbilliviruses with ClustalW (www.ebi.ac.uk/clustalw) (the primer sequences having been removed); phylogenetic studies were performed with the MEGA (version 4) package (www.megasoftware.net). This alignment indicated that this strain was very closely related to the virus that caused the epizootic in 1990 (Figure 2, **panel B**). Overall, 7 of the 10 dolphins (4 of 5 adults; 3 of 5 juveniles) examined during this study were positive for DMV by immunohistochemistry or PCR. PCR products spanning other viral genes are currently being generated and sequenced to develop a more detailed phylogenetic analysis of the virus currently circulating.

## Conclusions

These results show that DMV is again circulating in the Mediterranean population of striped dolphins. The new epizootic resembles the previous die-offs in 1990: both epizootics began in approximately the same region and at the same time and, according to the stranding patterns (Figure 1, **panel B**), have followed a similar course of infection. However, in the current epizootic, younger animals were apparently more severely affected by the disease. Although these results are based on stranded dolphins and stranding rates do not necessarily correlate directly with death rates (*9*), we are comparing data from the same area for the 2 epizootics. We are also aware that we used a surrogate of age (i.e., standard length) for these comparisons. However, standard length correlates with sexual maturity (*10*) and has been repeatedly used as proxy for age (*5*). Finally, diseased dolphins are still being detected in late 2007; more juveniles than adults are affected.

These observations further indicate that DMV did not persist as an enzootic infection in the Mediterranean striped dolphins after the 1990–1992 epizootic, that adult dolphins that had survived the first epizootic still had some immunity against the virus, and that unpredictable epizootics may recur. The relatively high density of striped dolphins, their gregarious behavior, and the decreasing level of specific immunity will likely favor the propagation of the virus among the entire Mediterranean population. For this reason, abnormally high die-offs might be expected in other areas of the Mediterranean Sea in the coming months, and measures should be taken to recover and analyze the carcasses.

Recurrent morbillivirus epizootics in marine mammals were described in harbor seals, *Phoca vitulina*, from northern Europe (*11*–*14*) and bottlenose dolphins, *Tursiops truncatus*, from the western Atlantic and Gulf of Mexico (*15*). Two major questions need to be answered concerning these phenomena: 1) what was the source of infection or reinfection, and 2) did environmental stress (e.g., pollutants, adverse weather, fisheries) precipitate the epizootics (*9*)? In the case of the Mediterranean striped dolphin, the answer to the first question is far from clear, but that both epizootics began in a region close to the Gibraltar Straits, where contacts with infected cetaceans of Atlantic origin could have occurred, is perhaps not coincidental. With regard to the second question, striped dolphins killed by the disease in 1990 had particularly high polychlorinated biphenyls levels, and water temperatures were abnormally high during the winter before the epizootic (*2*). The role of these environmental factors in the 2007 epizootic remains to be more fully investigated. Recurrent epizootics with high die-offs among all age classes will probably have a negative affect on the population dynamics of Mediterranean *S. coeruleoalba*.
